# Syphilitic Disease in the Spinal Column

**Published:** 1895-05-18

**Authors:** A. H. Tubby

**Affiliations:** Surgeon to the National Orthopædic Hospital; Surgeon to Out-Patients Evelina Hospital for Sick Children


### SYPHILITIC DISEASE IN THE SPINAL COLUMN.

By A. H. Tubby, M.S.Lond., F.R.C.S.Eng., Surgeon to the National Orthopædic Hospital; Surgeon to Out-patients Evelina Hospital for Sick Children.

In dealing with the causes of Pott's disease there is ample clinical evidence that syphilis gives rise to considerable posterior curvature following necrosis and caries.

In the cervical region, the part most frequently affected, numerous cases of syphilitic spinal disease are recorded in surgical literature. The frequent occurrence of disease in this part may be due to a spreading of syphilitic ulceration from the pharynx, but more often the process originates in the bodies of the vertebræ themselves.

A case is recorded, and the specimen figured, by Hilton[1] in "Rest and Pain." The disease is situated between the occiput and atlas, and between the atlas and axis. The probable history of the specimen is that "the man to whom it had belonged had been long the subject of syphilis, had suffered great pain in the neck, and that, after eating his dinner, his head fell forward upon the table, and he died instantly."

Another probable instance is recorded by Dr. Dunlop,[2] of Jersey. In this case, although the cause is not stated to have been syphilis, yet the occurrence of swellings in the buttock, chest, and back of hand simultaneously with disease in the first and second cervical vertebræ strongly point to tertiary specific disease. This suspicion is strengthened by the woman's age, 58 years, when she first suffered from pain in the neck and loins.

An instance occurring in the dorsal region is given by Mr. Howard Marsh.[3] A man, 45 years of age, came to St. Bartholomew's with tertiary syphilis, from which he had suffered severely at intervals for upwards of fifteen years. He now had several broken-down gummata on the skull, with severe hemicrania, and numerous syphilitic scars about his face, trunk, and limbs. He complained of severe nocturnal pains in his back, and said that his spine was becoming bent and so stiff that he could not stand upright. On examination, the dorsal curve of the spine was found to be considerably increased, so that the shoulders were very round and the head was bent forward. He rapidly improved on iodide of potassium, gradually increased to twenty grains three times a day, and the gummata were absorbed. He has had fresh outbreaks of tertiary syphilis since, and "each attack has left the spine more arched and more stiff, and when I saw him last he was unable to raise his head above the level of his lower dorsal vertebræ."

Reeves[4] mentions a case of syphilitic caries in a boy under his treatment at the London Hospital, who coughed up portions of vertebræ which had penetrated the lung.

As illustrating the incidence of syphilitic disease in the lumbar region of the spine, I may cite with advantage the following case of Fournier's.[5]—Aman, aged 56, tall and well developed, was admitted into the hospital St. Louis, in July, 1876. "He had been losing health for some time, and suffering from pains in the loins and lower limbs. Examination showed marked evidence of syphilis of long standing, *e.g.*, syphilitic sarcocele, ten cutaneous gummata, others in the muscles, and a gummatous ulceration of the great toe, also a macula on the thigh. In spite of treatment this man's condition soon became worse, and he died in October, 1876. *Post-mortem* examination. — In addition to the above lesions there were found characteristic cicatrices in the spleen, gumma of the fourth lumbar nerve, multiple and considerable lesions as of Pott's disease, affecting the lumbar vertebral column, especially the third, fourth, and fifth vertebræ of that region. These consisted of denudations of bones, thickening or destruction of the periosteal and ligamentous structures, sclerosing osteitis with caseous and purulent infiltration, almost complete destruction of the inter-vertebral fibro-cartilage, and a vast hollow mass in the lumbar column, also an abscess in each psoas muscle. A careful microscopical examination showed the deposit in these bones to be clearly gummata in various stages of degeneration; also there were gummatous modules in the nerves passing off from this region."

From the details of the case quoted above we can gather that syphilitic caries of the spine offers a close parallel to tubercular or traumatic caries. In all the varieties of Pott's disease we have corresponding symptoms. In syphilitic caries the following points are particularly striking: (1) It begins later in life than other forms of caries; (2) it affects the cervical region by preference, notably the atlas, axis, and third cervical vertebræ; (3) it is accompanied by rapid destruction of bone, to which, in some cases, repair succeeds very rapidly also; (4) when caries is present other signs of tertiary syphilis are well marked; (5) it occurs rarely in congenital syphilis.

Ridlon, writing on syphilitic spondylitis in children,[6] says that it differs in no way from syphilitic joint disease elsewhere, except that it is deeply situated, since it usually occurs about the anterior surface of the bodies. Its onset appears to be comparatively rapid; if in the dorsal region, kyphosis soon appears with a sharp angle and sweeping curves above and below. When the disease is well established it follows the rule of all syphilitic lesions, that is resolution or pus, and the abscess will undergo rapid absorption if the patient is placed under the influence of mercurials. He adds, spondylitis associated with joint disease in young children under three is more often syphilitic than tuberculous. The most common associated conditions are bone and joint disease elsewhere, and the least common are skin eruptions.

**Treatment.**—This must be conducted on the same principles as Pott's disease arising from other causes, special attention being devoted to the specific taint.

[1] Op. cit., p. 111. [2] Brit. Med. Journ., 1893, vol. ii., p. 1,380. [3] B. M. J., 1893, vol. ii., p. 793. [4] Op. cit., p. 133. [5] Annal de Dermat. and de Syph., Jan. 1881, and quoted by Barker, Syst. Surg., vol. ii., p. 421, [6] Trans. of Ortho. Assoc., vol. iv., p. 118.

